# Bacterial–fungal interactions and response to heavy metal contamination of soil in agricultural areas

**DOI:** 10.3389/fmicb.2024.1395154

**Published:** 2024-05-10

**Authors:** Jia Li, Qiwen Zheng, Jiangyun Liu, Shuwei Pei, Zhen Yang, Rentong Chen, Li Ma, Jingping Niu, Tian Tian

**Affiliations:** ^1^School of Public Health, Lanzhou University, Lanzhou, Gansu, China; ^2^Lanzhou Maternal and Child Health Care Hospital, Lanzhou, Gansu, China

**Keywords:** heavy metal contamination, soil microbial community, high-throughput sequencing, co-occurrence network, bacterial–fungal interactions, keystone taxa

## Abstract

**Introduction:**

Long-term heavy metal contamination of soil affects the structure and function of microbial communities. The aim of our study was to investigate the effect of soil heavy metal contamination on microorganisms and the impact of different heavy metal pollution levels on the microbial interactions.

**Methods:**

We collected soil samples and determined soil properties. Microbial diversity was analyzed in two groups of samples using high-throughput sequencing technology. Additionally, we constructed microbial networks to analyze microbial interactions.

**Results:**

The pollution load index (PLI) < 1 indicates that the area is not polluted. 1 < PLI < 2 represents moderate pollution. PLI was 1.05 and 0.14 for the heavy metal contaminated area and the uncontaminated area, respectively. Cd, Hg, Pb, Zn, and Cu were identified as the major contaminants in the contaminated area, with the contamination factors were 30.35, 11.26, 5.46, 5.19, and 2.46, respectively. The diversities and compositions of the bacterial community varied significantly between the two groups. Compared to the uncontaminated area, the co-occurrence network between bacterial and fungal species in the contaminated area was more complex. The keystone taxa of the co-occurrence network in the contaminated area were more than those in the uncontaminated area and were completely different from it.

**Discussion:**

Heavy metal concentrations played a crucial role in shaping the difference in microbial community compositions. Microorganisms adapt to long-term and moderate levels of heavy metal contamination through enhanced interactions. Bacteria resistant to heavy metal concentrations may play an important role in soils contaminated with moderate levels of heavy metals over long periods of time.

## 1 Introduction

In recent years, soil contamination with heavy metals has received widespread attention due to their high biological toxicity, non-biodegradability, and long-term accumulation in soil (Li et al., 2023). Anthropogenic activities are important causes of heavy metal contamination in soil, such as mineral exploitation, agricultural activities, industrial activities, and transportation (Zhang et al., 2023). It has been reported that industrialization has led to heavy metal contamination in many of China's agricultural soils, and the excessive levels of heavy metals in agricultural land have seriously hampered the development of food security in China (Cai and Yang, 2023). In addition, compared to single-heavy metal contamination, multiple heavy metal contamination is more complex and poses a threat not only to human health but also to ecological functions (Qi et al., 2022). Many studies have shown that heavy metals are detrimental to soil microorganisms, affecting their diversity, community structure, and function (Cong et al., 2014; Smidt et al., 2018; Xu et al., 2019). For example, the structure of soil microorganisms in lead (Pb), cadmium (Cd), and zinc (Zn) co-polluted soils has been found to be altered significantly, with Firmicutes being the most resistant to heavy metals (Fajardo et al., 2019). Shuaib et al. found that heavy metals, such as Hg, Cr, Pb, Mn, and As, impacted microbial communities in soil and led to the death of organisms (Shuaib et al., 2021). Other researchers showed that long-term heavy metal pollution affects the predicted functions of soil microbial communities (Li et al., 2020). Thus, the effects of heavy metals on microbial communities have received increasing attention.

Soil microorganisms play a key role in biogeochemical processes, pollutant degradation, and maintaining the balance of soil ecosystems (Wang W. et al., 2022; Qian et al., 2024). They can respond to changes in the external environment by adjusting their members, activity, and population composition (Ma et al., 2022; Li et al., 2024). More importantly, soil microorganisms are more sensitive to changes in heavy metals than plants and animals and have the potential to be used as biomarkers of soil environmental quality (Luo et al., 2019). In addition, heavy metals cannot be degraded, but mineralization by microorganisms can convert them into less toxic or less mobile states (Lin et al., 2023). It is necessary to study the survival strategies of microorganisms under heavy metal pollution.

The community composition of soil microorganisms is influenced not only by environmental factors but also by the interactions between microbial species (Du et al., 2023). Bacterial–fungal interactions are prevalent in various ecological environments (Arnold, 2022), and their interactions may be conducive to the adaptation of microbes to multiple heavy metal-contaminated soils. Although soil microorganisms have been studied extensively, the effects of bacterial–fungal interactions in heavy metal-contaminated soils are still poorly understood. In order to uncover the changes in the survival patterns of microorganisms in heavy metal-contaminated environments, it is imperative to explore the bacterial–fungal interactions.

Network analysis is a powerful method for revealing ecological interactions among microbial communities in various environments (Yu et al., 2020; Yun et al., 2021). In recent years, network analysis has been used more and more frequently in the field of soil microbial ecology (Creamer et al., 2016). The advantages of networks are derived from their capacity to extract novel information on ecological interactions, establishments, keystone taxa, and microbial interactions. Keystone taxa play an important role in sustaining community stability and regulating microbial interactions within the soil microbiome (Xun et al., 2021). Therefore, it is important to investigate the variation of keystone taxa under multimetal-contaminated conditions.

In the present study, we selected agricultural soils in Baiyin and Lanzhou cities of Gansu Province, China, as the study area. We investigated the effects of heavy metal-contaminated soils on microorganisms and constructed co-occurrence networks based on the amplicon sequence variant (ASV) levels for assessing the correlation and co-occurrence patterns between bacteria and fungi. This research will provide new insights into the survival strategies of microorganisms in heavy metal-contaminated soils. The objectives of this research were as follows: (1) bacteria and fungi in soil respond variably to heavy metal contamination; (2) bacterial–fungal interactions are influenced by heavy metal pollution; and (3) there are keystone taxa in the microbial co-occurrence network that contribute to the resistance of microbial communities to heavy metals.

## 2 Materials and methods

### 2.1 Study area and sample collection

**Figure 1:**
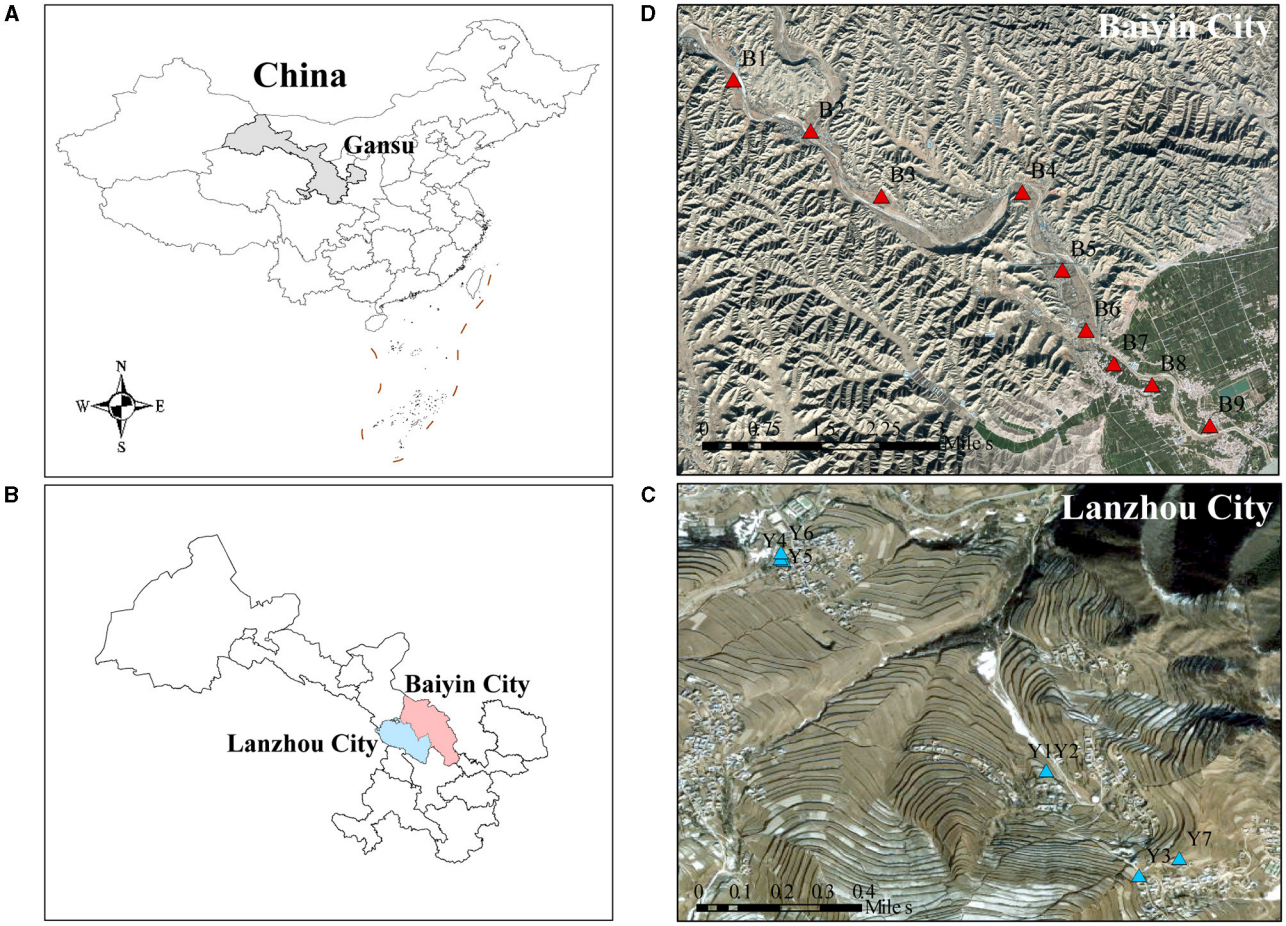
The location of sampling points in the contaminated and uncontaminated areas. **(A)** China, **(B)** Baiyin and Lanzhou are shown on the left. The geographical locations of the **(C)** contaminated and **(D)** uncontaminated sampling sites are shown on the right.

The research sites are located in Baiyin (36°29′N, 104°17′E) and Lanzhou City (35°46′N, 104°1′E), Gansu Province, northwestern China, where the soil type is gray calcareous soil (Figure 1). The heavy metal-contaminated soil samples were collected in the agricultural area along the Dongdagou stream, the main tributary of the Yellow River in Baiyin, which belongs to the transition zone from the middle temperate semi-arid zone to the arid zone that has a temperate continental climate. The local average annual rainfall is 220 mm. Due to the lack of precipitation, there is a local habit of using wastewater for irrigation (Nan and Zhao, 2000). Previous studies have shown that the average concentrations of heavy metals in water samples from the East Dagou River exceeded the maximum permitted levels of pollutants specified in the Chinese National Irrigation Water Quality Standards (GB5084-2005) (He et al., 2021; Wu et al., 2023). As a result, the site is typical of an area contaminated with multiple heavy metals. The non-heavy metal-contaminated soil samples were collected from an agricultural area south of Xinglong Mountain Nature Reserve in Yuzhong County, Lanzhou City, with a similar geography and climate to that of Baiyin. The local area soil is not contaminated with heavy metals.

Topsoil was collected in October 2021. According to the Technical Specification for Soil Environmental Monitoring (HJ/ST166-2004), at each sampling site, a piece of farmland with an area of about 10 × 10 m was randomly selected, and five sub-sampling sites were set up in each selected farmland using the five-point sampling method. A total of 16 samples were selected in this study: nine samples were collected from Baiyin and seven samples were collected from Lanzhou. Soil was collected with a wooden shovel and mixed well to a depth of ~20 cm with a sample weight >1 kg. Soil samples were sealed in polyethylene bags and sent to the laboratory within 24 h. One part of the soil was stored at −80°C for subsequent DNA extraction, and the other part was homogenized with a 2 mm mesh sieve to remove stones, roots, and other debris and stored at 4°C for soil property analysis.

### 2.2 Soil property analysis

The total phosphorus (TP) content was determined by the alkali fusion-Mo-Sb anti spectrophotometric method, available phosphorus (AP) was determined by the sodium hydrogen carbonate solution-Mo-Sb anti-spectrophotometric method, total nitrogen (TN) was determined by the Kjeldahl method, organic carbon (OC) was determined by the potassium dichromate titrimetric method, ammonium nitrogen (AMN) was determined by the indophenol blue colorimetric method, and nitrate nitrogen (NN) was determined by the ultraviolet spectrophotometry method. Microbial biomass carbon (MBC), microbial biomass nitrogen (MBN), and microbial biological phosphorus (MBP) were measured by the fumigation–extraction method.

Soil samples of ~0.5 g were digested with 2 ml HCl, 6 ml HNO_3_, and 2 ml HF in a microwave digestion system (Milestone ETHOS ONE). Then, inductively coupled plasma–mass spectrometry (ICP–MS, Agilent, United States) was used to determine the content of heavy metals (Mo, Cd, Sb, Cu, Zn, Hg, and Pb). Quality assurance/control procedures were performed using standardized reference materials (the Chinese Academy of Measurement Science) for each batch of samples (one blank and one standard).

### 2.3 Soil microbial DNA extraction and sequencing data processing

Total microbial genomic DNA was extracted from soil samples using the PowerSoil^®^ DNA Isolation Kit (MO BIO Laboratories, USA) according to the manufacturer's instructions. The quality and concentration of DNA were determined by 1.0% agarose gel electrophoresis and a GeneAmp^®^ 3 platform (ABI Inc., USA), and the sample was kept at −80°C prior to further use. The V4 region of the bacterial 16S rRNA gene was amplified using the primer pair 515F (5′-GTGCCAGCMGCCGCGGTAA-3′) and 806R (5′′-GGACTACHVGGGTWTCTAAT-3′) (Liu et al., 2015). The internal transcribed spacer (ITS) region of the fungal rRNA genes was amplified using the primer pair ITS1F (5′-CTTGGTCATTTAGAGGAAGTAA-3′) and ITS2R (5′ -GCTGCGTTCTTCAT CGATGC-3′) (Adams et al., 2013) by an ABI GeneAmp^®^ 9700 PCR thermocycler (ABI, CA, USA). The PCR reaction mixture included 4 μl of 5 × FastPfu buffer, 2 μl of 2.5 mM dNTPs, 0.8 μl of each primer (5 μM), 0.4 μl of FastPfu polymerase, 10 ng of template DNA, and ddH2O mixed to a final volume of 20 μl. PCR amplification cycling conditions were as follows: initial denaturation at 95°C for 3 min, followed by 30 cycles of denaturing at 95°C for 30 s, annealing at 55°C for 30 s, extension at 72°C for 45 s, single extension at 72°C for 10 min, and end at 4°C. All samples were amplified in triplicate. The PCR product was extracted by electrophoresis using 2% agarose gel. The concentration of PCR products was determined by NanoDrop spectrophotometer (ND-2000 Spectrophotometer, Thermo Fisher Scientific, USA).

Amplicons were gel-purified using an AxyPrep DNA Gel Extraction Kit (Axygen Biosciences, USA) and sequenced on an Illumina MiSeq platform at the Majorbio Bio-pharm Technology Co., Ltd. (Shanghai, China). The resulting sequences were processed by using the DADA2 plugin in the QIIME2 package for quality filtering, denoising, merging, and dereplication. Then, the sequences were clustered into amplified sequence variants (ASVs), which are considered more precise and comprehensive than operational taxonomic units (OTUs) (Callahan et al., 2017).

### 2.4 Construction of the co-occurrence network

We constructed bacterial–fungal interspecies co-occurrence networks using the “Hmisc” and “igraph” R packages based on Spearman's correlation coefficient. To reduce network complexity and facilitate the identification of the core soil community, thresholds were selected with absolute values of Spearman's correlation coefficient (*r*) of > 0.7 and Benjamini and Hochberg false discovery rate (FDR)-corrected *p*-values of < 0.05 (Barberan et al., 2012). Network images were generated using Gephi (version v 0.9.2), and network properties (i.e., average degree, average clustering coefficient, density, and modularity) were calculated. The topological roles of the nodes in the network are classified according to the threshold values of *Zi* (within-module connectivity) and *Pi* (among-module connectivity). Module hubs (*Zi* > 2.5), network hubs (*Zi* > 2.5 and *Pi* > 0.62), and connectors (*Pi* > 0.62) of the network were considered critical nodes and identified as keystone taxa.

### 2.5 Statistical analysis

The level of pollution caused by individual metals was evaluated using the contamination factor (CF), as shown in Equation (1). The level of contamination by multiple metals in the study area was assessed comprehensively using the pollution load index (PLI), as shown in Equation (2) (Bhuiyan et al., 2010). The calculation formula is as follows:


(1) $$\begin{array}{l} \text{CF}={\text{Metal}}_{\left[\text{Measured}\right]}/{\text{Metal}}_{\left[\text{Background}\right]} \end{array}$$

(2) $$\begin{array}{l} \text{PLI}={\left({\text{CF}}_{1}\;\times \;{\text{CF}}_{2}\;\times \;\cdot \cdot \cdot \;\times \;{\text{CF}}_{\text{n}}\right)}^{1/\text{n}} \end{array}$$

where Metal _[Measured]_ is the concentration of heavy metals in the soil samples, Metal _[Background]_ is the background value of heavy metals, and n is the number of heavy metals measured in this study. The values of 1 < CF < 3 indicate moderate pollution, 3 < CF < 6 indicates considerable high pollution, and CF > 6 indicates that pollution is at the highest level (Esmaeilzadeh et al., 2021). If PLI > 1, it is considered a contaminated area. The higher the value, the more severe the heavy metal pollution.

Alpha diversity indexes, including coverage, Chao1 richness, and Simpson index, were calculated using the “vegan” and “picante” R packages. The Mantel test was performed using “vegan” and “ggplot2” R packages to obtain the significance of Spearman's rank correlations between microbial communities and heavy metals. Correlation heat maps were calculated and plotted using the “psych” and “pheatmap” R packages, respectively. Principal component analysis (PCA) was used to evaluate the influence of heavy metal pollution on the structure of microbial communities. The Wilcoxon Mann–Whitney test with the IBM SPSS 26.0 software was performed to compare alpha diversity indexes and pollution load index (PLI) between groups, and the *P*-value of < 0.05 was deemed to be statistically significant.

## 3 Results

### 3.1 Soil heavy metal concentrations

**Figure 2:**
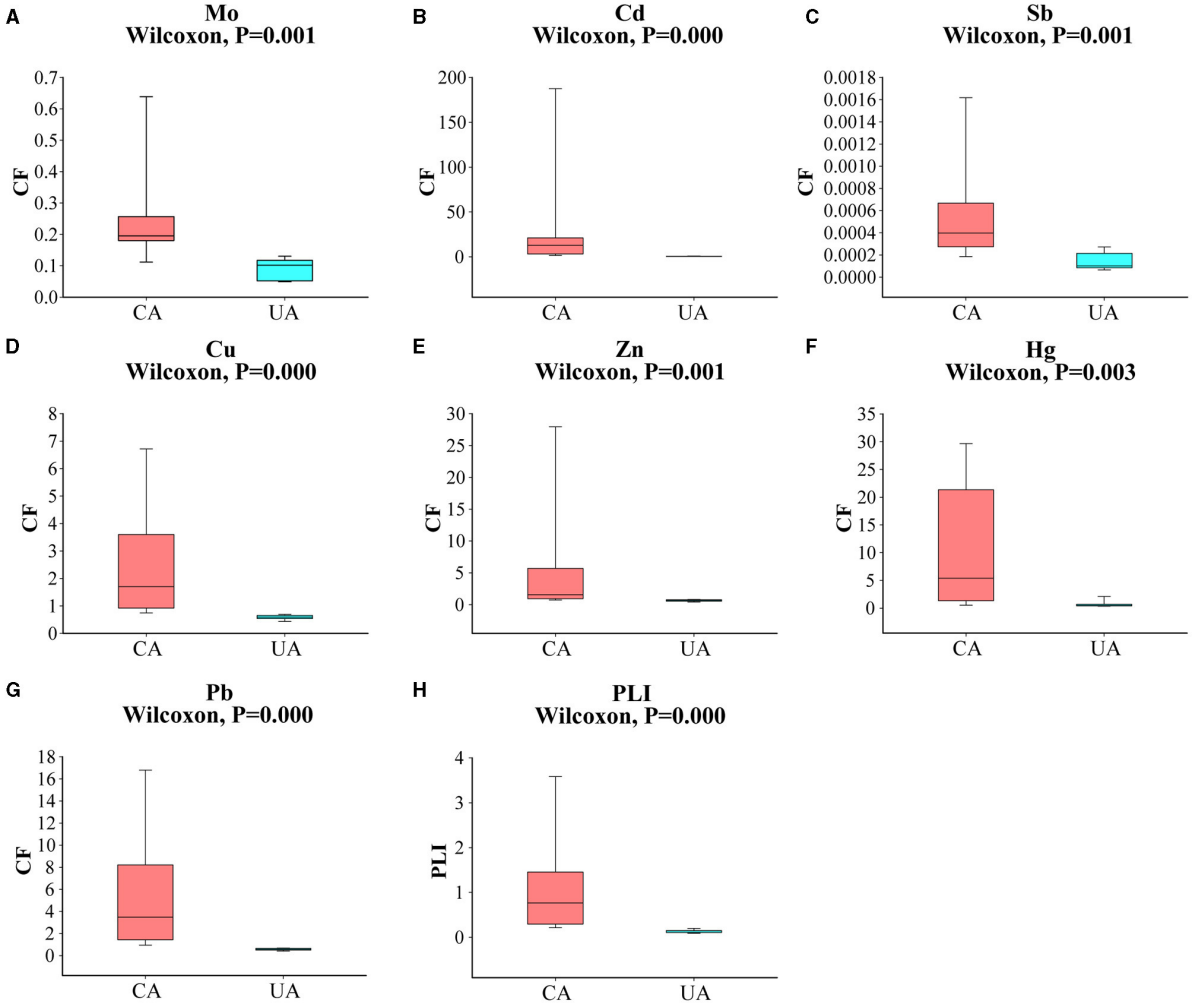
A comparison of heavy metal pollution factors between contaminated and uncontaminated areas **(A–G)**; **(H)** a comparison of pollution load indices between contaminated and uncontaminated areas.

The main soil type in the study area is gray calcareous soil, which is characterized by a low content of organic matter and nutrients (Yang et al., 2017). Since the level of pollution caused by heavy metals varied significantly, we classified the sampling sites according to the concentration of heavy metals. Baiyin soil samples were defined as “contaminated area” and Lanzhou soil samples were defined as “uncontaminated area”. As shown in Figure 2, the CF values of Mo, Cd, Sb, Cu, Zn, Hg, and Pb were significantly different in the contaminated and uncontaminated areas (Wilcoxon, *P* < 0.05). Previous studies have confirmed that the sources of pollution in the contaminated area were mainly metallurgical plant dust and slag heap, and the level of pollution gradually decreased with the distance from the main sources (Wang et al., 2015). In addition, there was a local habit of using wastewater for irrigation. In the present study, large volatility was observed in the distribution of heavy metal content in contaminated areas, which is largely due to anthropogenic activities (Yue et al., 2020). Overall heavy metal contamination levels in the two groups of areas were significantly different (Wilcoxon, *P* < 0.05). The level of heavy metal in the contaminated area was significantly higher than those in the uncontaminated area. The average PLI was 1.05 in the contaminated area and 0.14 in the uncontaminated area. PLI > 1 indicated moderate pollution, and PLI < 1 indicated an uncontaminated level. The levels of Mo and Sb in all soil samples did not exceed local background values. Sb was the lowest heavy metal that occurred at the agricultural soil sites in both study areas, reaching concentrations of 12.50 and 3.26 ng g^−1^, respectively (Supplementary Table S1). We considered that the Sb content in the soil at the sampling sites was very low and that the effect on the soil microbial communities would be relatively minimal. The contents of Cd, Cu, Zn, Hg, and Pb were higher than the local background values in the contaminated area and lower than the local background values in the uncontaminated area. Specifically, the Cd concentrations were, on average, 50 times higher in the contaminated area than in the uncontaminated area. Compared with Cd concentrations, other heavy metals showed less concentration differences (<16 times) in these two areas. The second highest concentration in the contaminated area was Hg, with an average CF value of 11.26, indicating the highest level.

The physicochemical properties of the soil samples are shown in Table 1. Microbial biomass carbon (MBC), microbial biomass nitrogen (MBN), total nitrogen (TN), organic carbon (OC), and nitrate nitrogen (NN) were significantly higher in the uncontaminated than in the contaminated area (Wilcoxon, *P* < 0.05). Microbial biological phosphorus (MBP), water content (WC), total phosphorus (TP), available phosphorous (AP), and ammonium nitrogen (AMN) levels were not significantly different between the two groups (Wilcoxon, *P* > 0.05).

**Table 1:** Soil physicochemical properties.

**Parameter**	**CA**	**UA**	***P*-value**
MBC	115.58 ± 29.4	174.16 ± 35.36	0.000
MBN	15.85 ± 3.29	20.73 ± 5.68	0.000
MBP	8.10 ± 2.66	8.08 ± 3.94	0.611
WC	14.42 ± 2.71	14.94 ± 1.56	0.187
TP	0.50 ± 0.45	0.35 ± 0.11	0.574
AP	70.73 ± 56.26	45.73 ± 24.43	0.275
TN	0.93 ± 0.25	1.40 ± 0.34	0.000
OC	13.58 ± 3.63	18.93 ± 4.94	0.000
AMN	0.79 ± 0.16	1.37 ± 1.29	0.274
NN	2.11 ± 0.78	4.66 ± 3.33	0.006

*P*-value: the significance of physicochemical properties between contaminated and uncontaminated area soils compared with the two-tailed Wilcoxon rank-sum test.

### 3.2 Richness and diversity of soil microbial communities

Based on ASV data, three α diversity indexes (coverage, Chao1, and Simpson) were used to evaluate the coverage, richness, and evenness of the soil microbial communities (Table 2). There was no significant difference for all indexes of bacteria and fungi between contaminated and uncontaminated areas except the Simpson index for bacteria. It was shown that heavy metal contamination did not change the abundance and diversity of bacteria and fungi but had an effect on the homogeneity of the bacterial communities. Moreover, the average coverage for bacteria and fungi was 0.980 and 1.000, respectively, which indicated that the current sequencing depth captured bacteria and fungi well. The average values of the Chao1 index for bacteria and fungi were 1,549.12 and 505.69, respectively. The abundance of bacteria in the soil was much higher than that of fungi, which is consistent with the results of previous studies, indicating that bacteria dominate the biomass and diversity of soil microorganisms (Deng et al., 2018).

**Table 2:** The α index of soil microbial communities.

**Sample**	**Coverage**		**Chao1**		**Simpson**	
	**Bacteria**	**Fungi**	**Bacteria**	**Fungi**	**Bacteria** ^*^	**Fungi**
CA	0.980 ± 0.008	1.000 ± 0.000	1,538.09 ± 385.44	486.04 ± 81.08	1.00 ± 0.00	0.97 ± 0.01
UA	0.979 ± 0.001	1.000 ± 0.000	1,563.29 ± 86.17	530.96 ± 94.65	0.99 ± 0.01	0.97 ± 0.02

Statistical differences between contaminated and uncontaminated areas were analyzed by a two-tailed Wilcoxon rank-sum test.

* Indicates *P* < 0.05.

**Figure 3:**
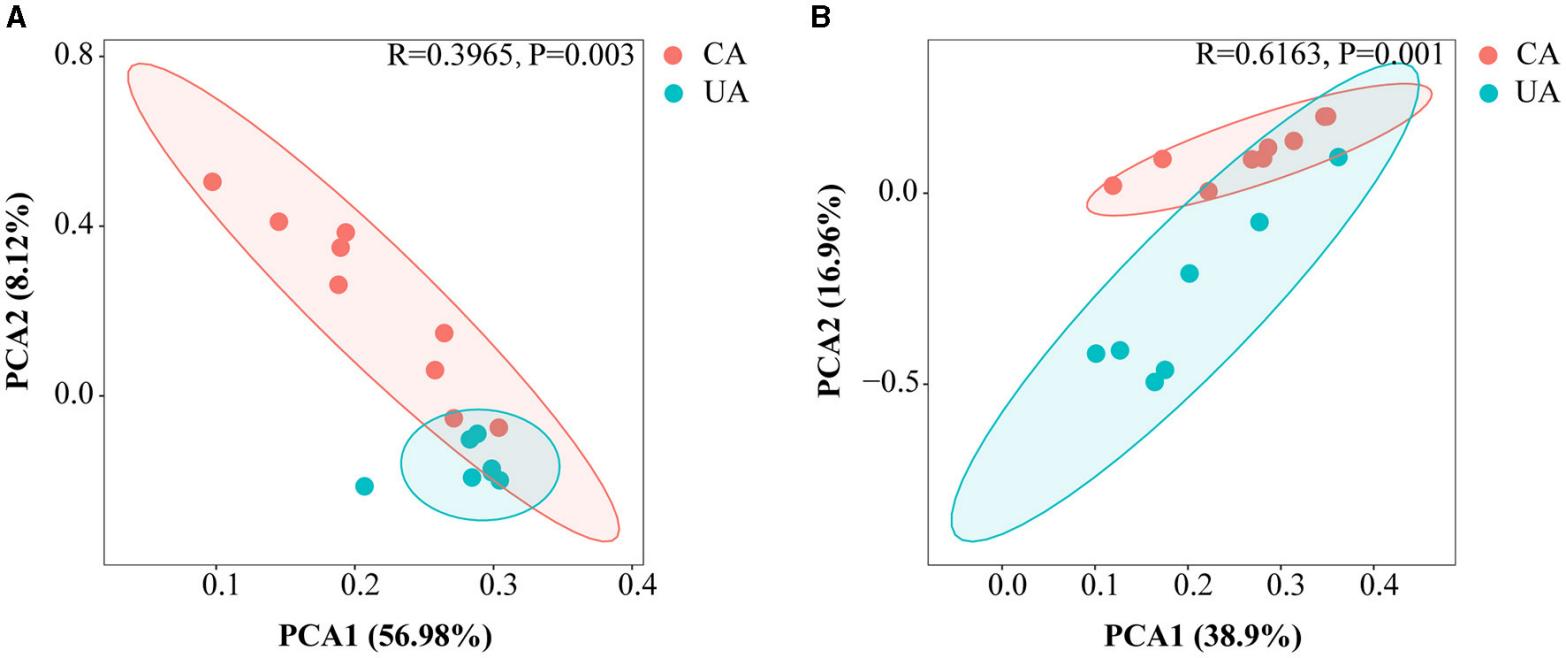
Principal component analysis based on the Bray–Curtis distance showing differences in microbial community compositions among differences in microbial community compositions at the ASV level. **(A)** The bacterial community **(B)** the fungal community.

Principal component analysis (PCA) was performed to estimate the β-diversity of soil microorganisms. As shown in Figure 3, the PCA1 axis of bacteria and fungi accounted for about 56.98 and 38.90% of the total variation, respectively. The PCA2 axis accounted for about 8.12 and 16.96%, respectively. The PCA1 and PCA2 axes accounted for a total of 65.10 and 55.86%, respectively, and the two major axes could explain most of the variation. In addition, analysis of similarity based on the Bray–Curtis distances (ANOSIM) showed significant differences in the distribution of bacterial and fungal communities between contaminated and uncontaminated areas (*R* = 0.3965, *P* = 0.003 and *R* = 0.6163, *P* = 0.001).

**Figure 4:**
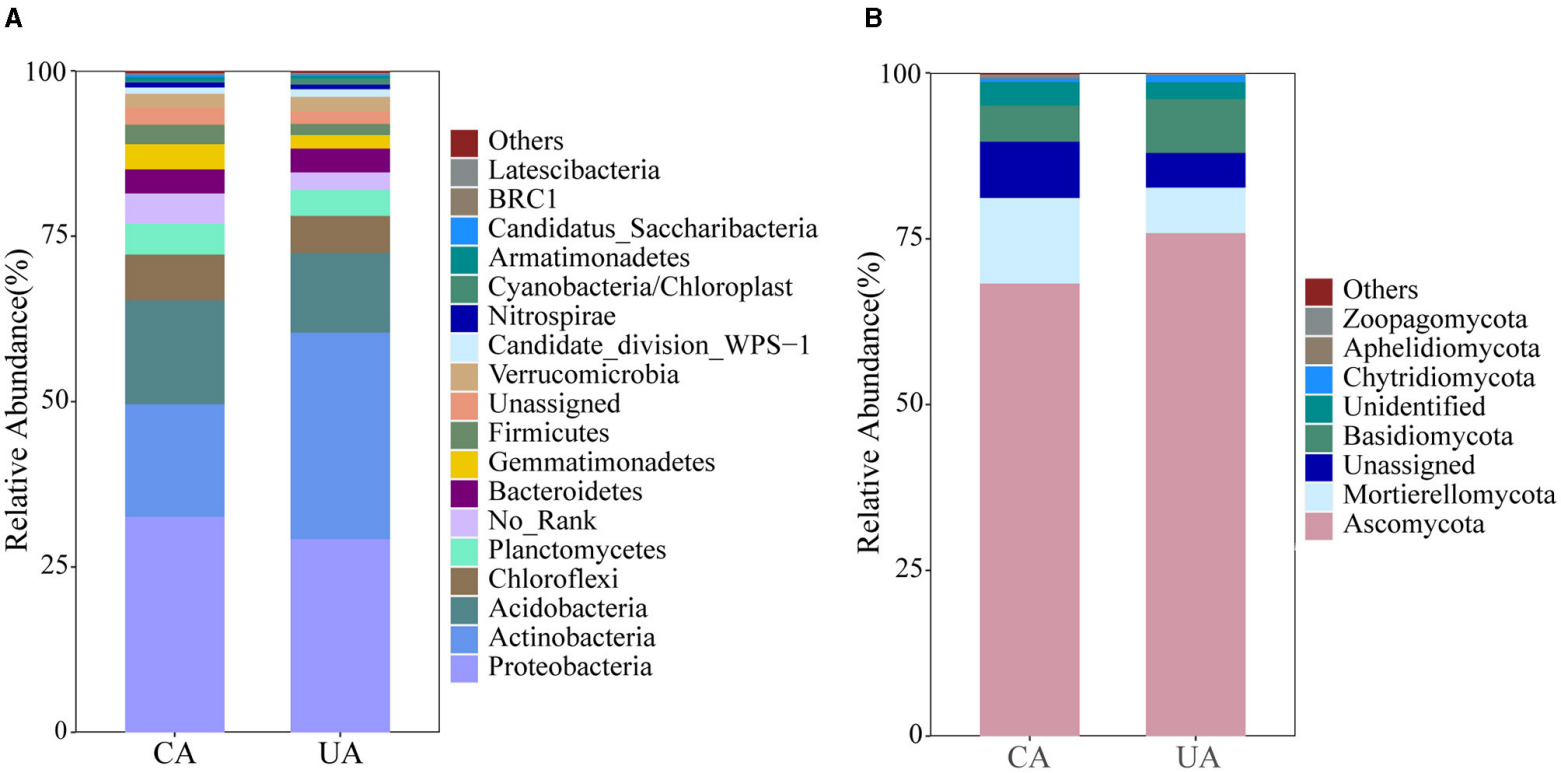
Community composition of soil bacteria **(A)** and fungi **(B)** at the phylum level.

The population composition of bacteria and fungi at the phylum level is shown in Figure 4. A total of 11,437 bacterial ASVs and 4,254 fungal ASVs were identified in the contaminated and uncontaminated areas. In total, 27 bacterial and 16 fungal phyla were identified. Differences exist in the distribution of microbial communities in soils with various levels of contamination. Proteobacteria, Actinobacteria, and Acidobacteria were the top three abundant phyla in the bacterial community. Actinobacteria showed significant preferences for the contaminated (17.02%) and uncontaminated (31.04%) areas (*P* < 0.001) (Supplementary Figure S1). Planctomycetes and Gemmatimonadetes were higher in the contaminated area than in the uncontaminated area.

A majority of fungal ITS gene sequences were associated with the phyla Ascomycota, Basidiomycota, and Mortierellomycota, which represented 86.57 and 90.67% of the total sequences in the contaminated and uncontaminated areas, respectively. Specifically, proportions of Ascomycota were much higher than other fungal phyla in both areas, with 65% in the contaminated and 75% in uncontaminated areas. The abundances of Mortierellomycota and Chytridiomycota were significantly different (*P* < 0.001) (Supplementary Figure S1). These results together suggested that heavy metal contamination has a strong impact on the soil microbial community structure.

### 3.3 Effects of environmental factors on microbial community composition

**Figure 5:**
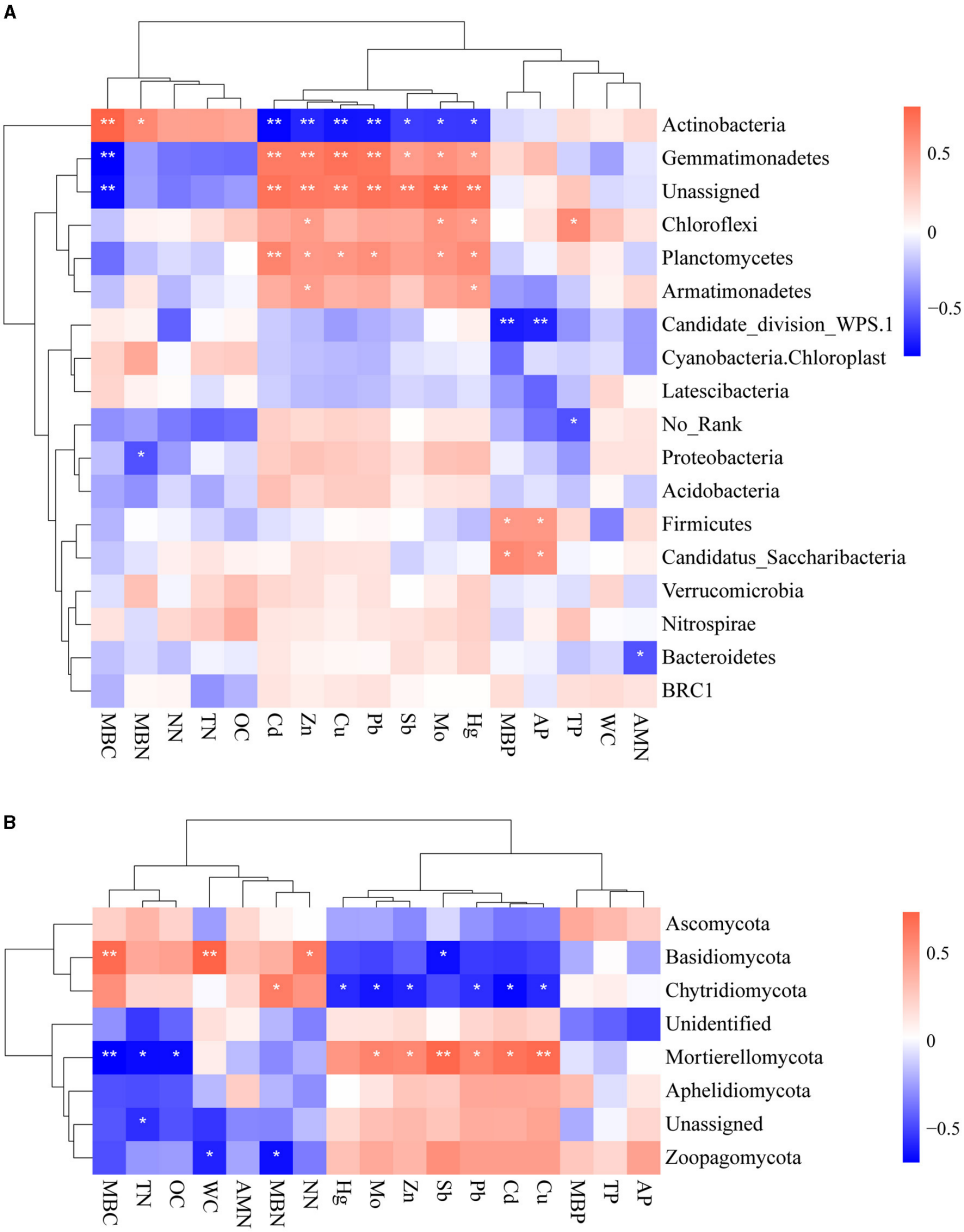
Spearman rank correlations between environmental factors and bacterial **(A)** and fungal community **(B)** (**0.001 < *p* < 0.01 and *0.01 < *p* < 0.05).

We studied the correlations between environmental factors and microbial taxa based on their relative abundance at the phylum level to gain an insight into how environmental factors affect the composition of microbial communities (Figures 5A, B). We observed mainly positive correlations between heavy metals and bacterial abundance. Zn and Hg concentrations showed uniformly positive associations with the relative abundance of bacteria Gemmatimonadetes, Chloroflexi, Planctomycetes, and Armatimonadetes. Gemmatimonadetes were notably positively associated with all heavy metals. Actinobacteria was negatively associated with all heavy metals. It demonstrated that Actinobacteria is sensitive to heavy metals, consistent with the above results. It is found that different types of physicochemical properties also have different effects on bacterial communities: MBC was positively correlated with Acidobacteriota and negatively correlated with Gemmatimonadetes, while MBP and AP were positively correlated with Firmicutes. Furthermore, several studies have reported that the phylum Proteobacteria was found to be abundant in many environments with high heavy metal content (Zhu et al., 2013; Chauhan et al., 2017; Zuo et al., 2023). Nonetheless, there was no significant correlation between Proteobacteria and heavy metals in this study. This might be attributed to the higher resistance of Proteobacteria to heavy metals.

Mo, Zn, Pb, Cd, and Cu were positively correlated with Mortierellomycota and negatively correlated with Chytridiomycota. It is evident that Sb was negatively correlated with Basidiomycota, which suggested its sensitivity to varied concentrations of Sb. Zoopagomycota was significantly associated only with physicochemical properties. MBC, TN, and OC had significantly negative effects on Mortierellomycota, while MBC, WC, and NN had significantly positive impacts on Basidiomycota. MBN was positively associated with Chytridiomycota and negatively associated with Zoopagomycota.

**Figure 6:**
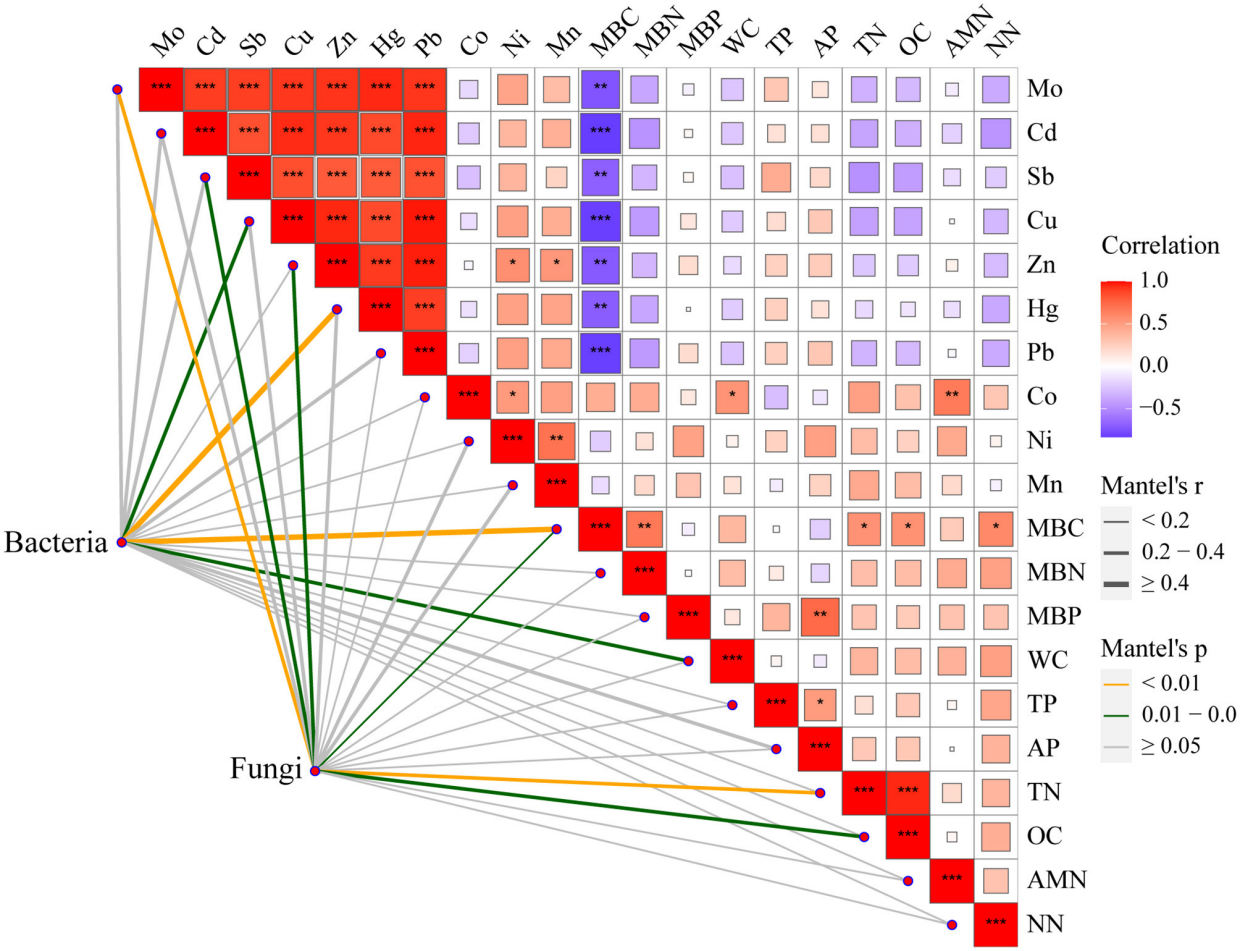
Spearman rank correlations between environmental factors and microbial community composition. ***p < 0.001, **0.001 < *p* < 0.01, and *0.01 < *p* < 0.05.

Moreover, the Mantel test based on ASV data showed a strong correlation between heavy metals, which might be attributed to the reason that these heavy metals come from the same source of contamination (Figure 6). In addition, MBC was negatively correlated with the above heavy metals and positively correlated with MBN, TN, OC, and NN. Cu, Hg, MBC, and WC had significant effects on the entire bacterial community. Mo, Cd, Sb, Zn, MBC, TN, and OC had significant effects on the entire fungal community. These results indicated that Cu plays an important role in altering the bacterial community after long-term contamination (Li et al., 2014).

### 3.4 Co-occurrence patterns of microbial communities

**Figure 7:**
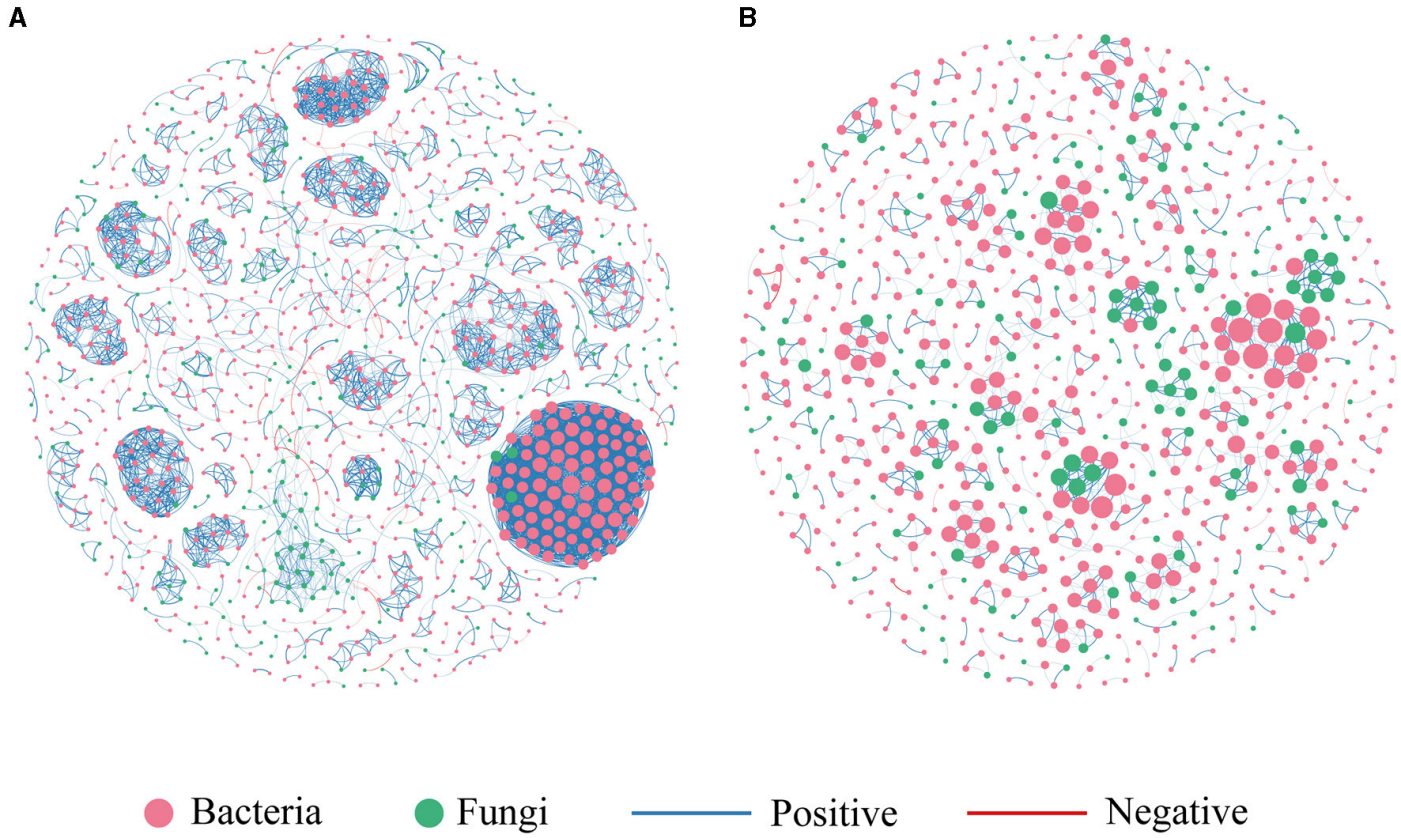
Co-occurrence network of microbial communities. **(A)** Contaminated areas **(B)** uncontaminated areas.

We constructed two co-occurrence networks at the ASV level for the contaminated and uncontaminated areas in order to identify associations between bacterial and fungal communities (Figure 7). The topological properties of the microbial association network are summarized in Table 3. Heavy metal pollution strengthened the connectivity, complexity, and degree of clustering of co-occurrence networks indicated by the increased average degree and density, respectively. It has been observed that complex networks with higher connectivity were more stable than simple networks with lower connectivity (Santolini and Barabási, 2018). In the two co-occurrence networks, the increase in nodes and edges of contaminated areas also implied that potential microbial interactions were closer in polluted soils. The node proportion of the fungal community was obviously lower than that of the bacterial community. This could be attributed to the highest bacterial diversity. Moreover, positive associations were abundant in all the two co-occurrence networks, which indicated that most of the bacterial–fungal interactions were primarily symbiotic.

**Table 3:** Topological characteristics of bacterial and fungal co-occurrence networks.

**Topological properties**	**CA**	**UA**
Nodes	1,242	782
Bacterial nodes (%)	77.78%	77.75%
Fungi nodes (%)	22.22%	22.25%
Edges	5,455	1,136
Positive edges (%)	98.64%	97.98%
Negative edges (%)	1.36%	2.02%
Average degree	8.784	2.905
Average clustering coefficient	0.663	0.846
Density	0.007	0.004
Modularity	0.775	0.975

**Figure 8:**
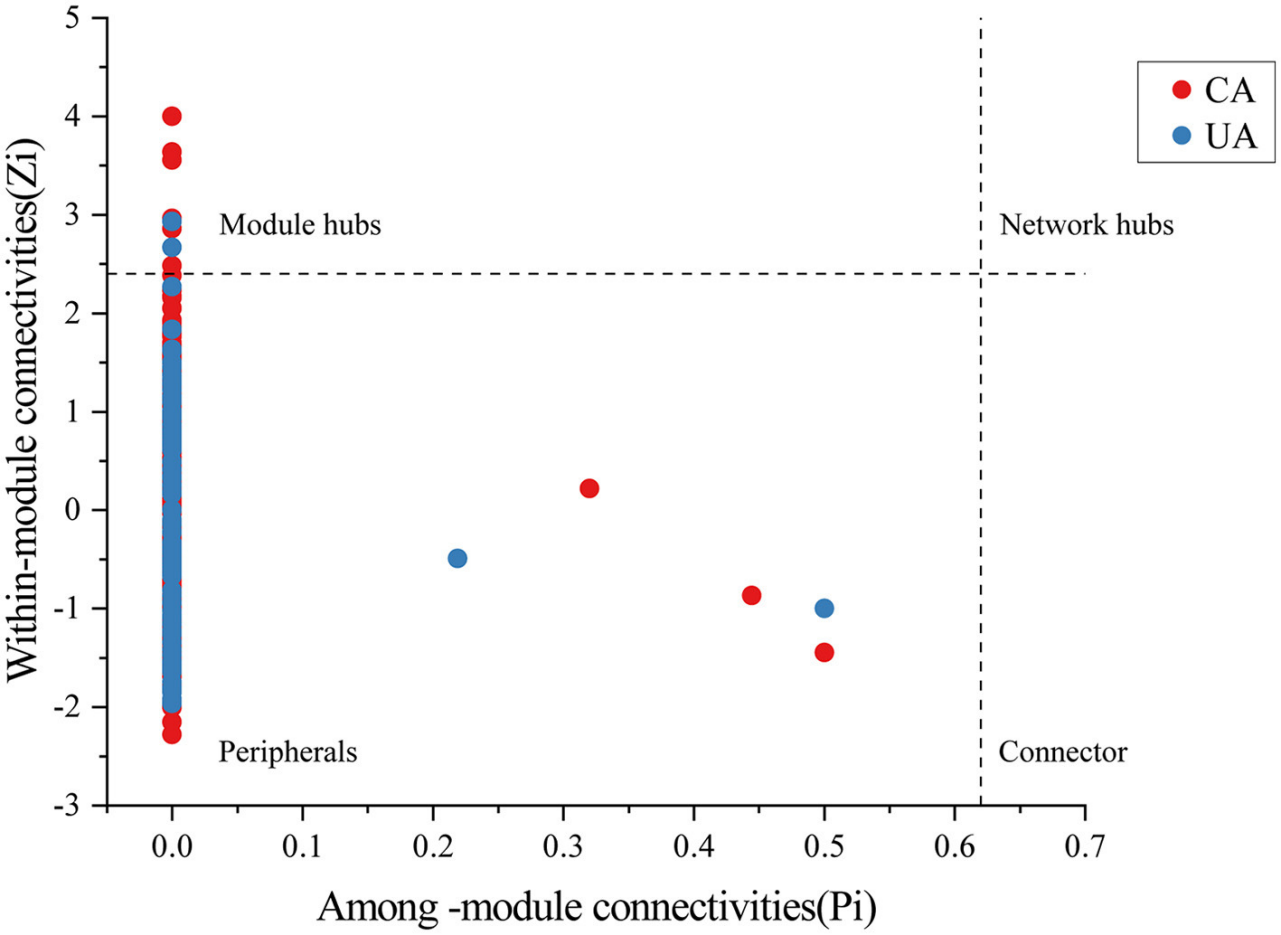
The analysis of bacterial and fungal ASV levels with network hubs and module hubs' role in network characterization.

Moreover, important taxonomic groups in the co-occurrence networks were extracted according to the values of *Zi* (within-module connectivity) and *Pi* (among-module connectivity) (Figure 8). Specifically, a total of seven and two ASVs were confirmed as the keystone taxa in the contaminated (five bacterial taxa and two fungal taxa) and uncontaminated (two bacterial taxa) areas, respectively. The keystone taxa of bacteria in the contaminated area were Chloroflexi, Armatimonadetes, Proteobacteria, and Acidobacteria, and the keystone taxa of fungi were all Ascomycota (Supplementary Table S2). The only keystone taxa in the uncontaminated area were bacteria, Verrucomicrobia, and Bacteroidetes (Supplementary Table S3). More keystone taxa were found in contaminated areas than in uncontaminated areas, which indicated that microorganisms will resist heavy metal stress through enhanced interactions. In addition, many of the bacterial keystone taxa are low-abundance colonies, suggesting that low-abundance bacteria may also play an essential role in the interaction of colonies to help resist heavy metal stress.

**Figure 9:**
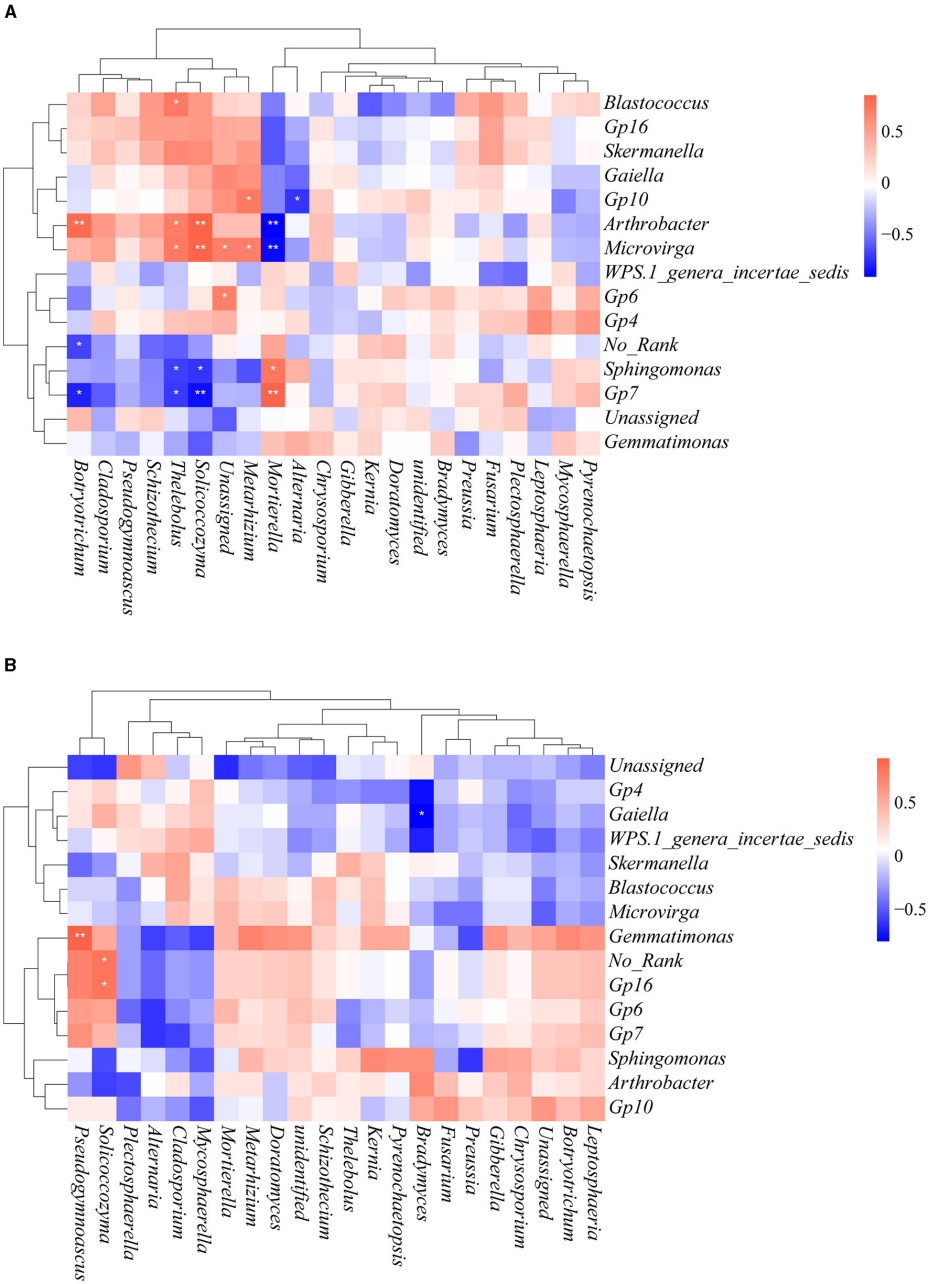
Spearman rank correlation between bacteria and fungi at the genus level. Only genera with relative abundance > 10% are shown. **(A)** Contaminated areas **(B)** uncontaminated areas (**0.001 < *P* < 0.01 and *0.01 < *P* < 0.05).

To gain further insights into the effects of heavy metal contamination on bacterial–fungal interactions, Spearman's correlation analysis was performed by selecting bacteria and fungi with relative abundance >10% at the genus level (Figure 9). There were more bacterial–fungal interactions in the contaminated area than in the uncontaminated area, which is consistent with the results of the co-occurrence network analysis. We observed that many of the fungi in the contaminated area were positively correlated with *Arthrobacter* and *Microvirga* but negatively correlated with *Sphingomonas* and *Gp7*. Interestingly, the interactions of *Mortierella* with these bacterial genera are in contrast to the above fungal genera. In the uncontaminated area, *Pseudogymnoascus* and *Solicoccozyma* were positively correlated with *Gemmatimonas* and *Gp 16*, respectively. *Bradymyces* was negatively correlated with *Gaiella*. These results illustrated that heavy metal contamination altered the interactions among soil microorganisms.

## 4 Discussion

### 4.1 The response of microbial communities to heavy metal pollution

Numerous studies have shown that heavy metal contamination reduces the diversity of soil microorganisms (Smidt et al., 2018; Zhang M. et al., 2022). However, in the present study, only the bacterial Simpson index was significantly different between the contaminated and uncontaminated areas. It is already known that bacteria are more sensitive than fungi in the face of heavy metal stress (Yang et al., 2023), which might be the reason for the difference in the bacterial Simpon index and no difference in fungi in this study.

On further comparing the differential species of the two groups of soil microorganisms, we observed that heavy metal contamination increased the abundance of Planctomycetes and Gemmatimonadetes but decreased the abundance of Actinobacteria. The results showed that Planctomycetes and Gemmatimonadetes were resistant to heavy metals and were able to survive in heavy metal-contaminated soils. Consistent with previous research, Actinobacteria were sensitive to heavy metals, which reduced their abundance significantly (Pan et al., 2020). In addition, Proteobacteria have been confirmed by many researchers as resistant to metal microorganisms and dominate in heavy metal-contaminated soils, which might be related to their complex livelihoods and the ability to degrade various complex organic molecules (Pereira et al., 2014). In this study, the abundance of Proteobacteria was higher in the contaminated area than in the uncontaminated area, but there was no significant difference between the two groups. This difference might be due to its relatively high resistance to some heavy metals and ability to live in extreme environments (Zhao et al., 2019; Yan et al., 2020). Therefore, slight contamination was unable to cause significant variation in its abundance.

Ascomycota was the highest relatively abundant among the fungi. Extensive degradation and metabolic properties and strong survival in a variety of habitats enable Ascomycota to rapidly adapt its community structure to its environment after exposure to heavy metal stress (Lin et al., 2019). Heavy metal pollution increased the abundance of Mortierellomycota and decreased the abundance of Basidiomycota and Chytridiomycota, indicating that Mortierellomycota is resistant to heavy metals, while Basidiomycota and Chytridiomycota are sensitive to heavy metals. As reported by previous studies, Gemmatimonadetes and Actinobacteria were correlated to Sb (Huang et al., 2023). The same results were observed in the present study. Considering the content of Sb was very low in both groups, we suggested that these species may be sensitive to Sb levels. In addition, Basidiomycota only correlated with Sb in heavy metals, and there may be suitable colonies to be biomarkers for Sb. However, the interaction between fungi and Sb merits further investigation (Wang W. et al., 2022).

Bacteria and fungi have different degrees of resistance to different levels of heavy metal pollution, as well as different abilities to accept and degrade heavy metals (Guo et al., 2017). Correlation analysis of environmental factors and microorganisms showed that bacteria and fungi responded differently to different environmental factors. Physicochemical properties and heavy metals had mostly contrasting effects on the same species. Among the bacteria, only Actinobacteria were negatively correlated with heavy metals, indicating that Actinobacteria was sensitive to heavy metals and that low concentrations of heavy metal pollution can reduce its abundance. Previous research has demonstrated that AP and AN had a strong influence on fungal communities in rhizosphere soil and bulk soils, while soil moisture contributed significantly to determine the structure of fungal communities in wetland soils (Onufrak et al., 2019; Wang Y. et al., 2022). In the present study, environmental factors cluster differently in fungi compared to bacteria. WC and AMN were not significantly different in the two groups but clustered together with the physicochemical properties that were significantly different. The reason for this result might be the relatively large contribution of these two environmental factors in driving changes in fungal communities.

The relationship between metallic elements indirectly reflects whether the elements have the same origin (Zhang and Sun, 2018; Jie et al., 2021). The higher the correlation between elements, the greater the possibility that they have the same source. In this study, there was a strong correlation between heavy metals, which might be due to the local habit of using wastewater to irrigate agricultural fields. Simultaneously, MBC showed a significant correlation with the above heavy metals as well as MBN, TN, OC, and NN. It has been reported that heavy metal contamination may alter soil chemistry to some extent (Shen et al., 2022). Therefore, it can be inferred that heavy metal contamination causes variations in MBC, which in turn affects other soil physicochemical properties. The uneven distribution of heavy metal Cd content and obvious spatial variation demonstrated that soil disturbance is mainly caused by anthropogenic activities (Zhao et al., 2020). Moreover, Cd was the most contaminated heavy metal in the contaminated area and is the most serious toxic metal threatening food safety and agricultural sustainability in China (Zhao et al., 2014).

### 4.2 The effects of heavy metal pollution on the co-occurrence patterns of microbial communities

Co-occurrence networks can be used to understand the potential ecological relationships among microbial communities. In this study, the average PLI value of the contaminated area was 1.05, indicating moderate pollution. Meanwhile, heavy metal contamination in the contaminated area was observed for a long term. The average PLI value of the uncontaminated area was 0.14, indicating uncontaminated levels. The results show that the co-occurrence network of the heavy metal contamination group was more intensive, with more nodes and higher co-occurrence number, connectivity, and stability. Previous research has shown that microorganisms tend to adapt to heavy metal pollution and increase their diversity and abundance accordingly if contamination continues for a long time (Bourceret et al., 2016). Studies have also revealed that microbial community function may be higher in heavy metal-contaminated soils (Singh et al., 2019). The above results indicated that the low levels of heavy metal pollution in the long-term may lead to improved adaptation of microorganisms to the environment and more efficient transfer of resources.

Previous studies have confirmed that moderate heavy metal contamination can enhance microbial interactions (Zhang X. et al., 2022). In this study, there were seven keystone taxa in the heavy metal-contaminated area and more than two in the uncontaminated area. Keystone taxa play a key role in the co-occurrence network, and an increase in the number of taxa further indicated the increased stability of the bacterial–fungal co-occurrence network in the heavy metal-contaminated areas (Xu et al., 2023). The species of keystone taxa in the contaminated and uncontaminated areas were different, further suggesting that heavy metal contamination could alter soil microbial interactions. Both keystone taxa identified in the uncontaminated area were bacteria, while five bacterial taxa and two fungal taxa were identified in the contaminated area. These fungi belonged to the Ascomycota phylum, which had the largest relative abundance. It has been shown that fungi in heavy metal-contaminated soils are more resistant to heavy metals than bacteria, and Ascomycetes had the highest resistance to heavy metals (Frossard et al., 2017; Zeng et al., 2020). In this study, heavy metal pollution increased the number of fungal keystone taxa. We hypothesized that fungi can increase tolerance to heavy metals by enhancing microbial interactions. The bacterial keystone taxa in the contaminated area were Chloroflexi, Armatimonadetes, Proteobacteria, and Acidobacteria. Among them, Chloroflexi was significantly positively correlated with Zn, Mo, and Hg. Armatimonadetes was positively correlated with Zn and Hg. The other two bacterial taxa had no significant correlation with heavy metals in this study. However, Proteobacteria was recognized as the tolerant phylum to heavy metals, and Acidobacteria was also been observed to be positively correlated with heavy metal concentrations (Pan et al., 2020). The keystone taxa Acidobacteria phylum at the genus level was *GP17*. It has been verified that the relative abundance of the genus *GP17* was higher in heavy metal-contaminated soils than in uncontaminated soils (Zhang M. et al., 2022). The keystone taxa in uncontaminated areas were Verrucomicrobia and Bacteroidetes, which were not significantly correlated with heavy metal concentrations in this study. However, previous studies showed that these two bacteria phyla were significantly negatively correlated with Zn, Pb, and Cr (Li et al., 2020). These results indicated that species with high tolerance to heavy metals will dominate the microbial interactions and play the role of keystone taxa in heavy metal-contaminated soils. Research has shown that a more stable microbial co-occurrence network would facilitate nutrient redistribution (Mo et al., 2022) and the stability of ecosystem function (Yuan et al., 2021). In addition, the relative abundance of keystone taxa at the genus level was low in both groups. This finding suggested that some low-abundance fungi could be keystone members in the soil microbiome and might play more critical roles in maintaining ecological stability and structuring fungal communities than some abundant genera. Moreover, the mechanism of the role of keystone taxa in the soil bacterial–fungal ecological network needs to be further explored. In conclusion, moderate-heavy metal pollution may contribute to the survival of microorganisms. Interspecific interactions between bacteria and fungi were a key element in the evolution of microbial communities in heavy metal-contaminated soil ecosystems (Chun et al., 2021).

Then, the interactions between soil bacteria and fungi were further analyzed (Figure 9). There were more bacterial–fungal interactions in the contaminated area than in the uncontaminated area, suggesting that heavy metals altered the interactions between soil microorganisms, which was consistent with the results of the co-occurrence network analysis. The correlated bacteria with fungi at the phylum level were Proteobacteria, Acidobacteria, Actinobacteria, Ascomycota, Basidiomycota, and Mortierellomycota in the contaminated area. The correlated bacteria with fungi at the phylum level in the uncontaminated area were Actinobacteria, Gemmatimonadetes, Ascomycota, and Basidiomycota. It has been documented that both Acidobacteria and Proteobacteria are the dominant phyla in heavy metal-contaminated areas (Jiang et al., 2021). In uncontaminated soil, these two bacterial taxa were not significantly correlated with fungi, whereas they were significantly correlated with fungi in contaminated soil. Bacterial–fungal interactions may enhance their resistance to heavy metals.

## 5 Conclusion

In this article, the contaminated area was mainly contaminated with the Cd, Cu, Zn, Hg, and Pb. The abundance and diversity of bacterial and fungal communities in the co-contaminated soils with multiple heavy metals did not change significantly, but the community distribution had significant changes. The relative abundance of Proteobacteria increased, whereas Actinobacteria showed the opposite trend in the case of heavy metal contamination. Ascomycota was the highest relatively abundant fungus among all study sites, much greater than that in other groups. Cu, Hg, MBC, and WC had significant effects on the entire bacterial community. Mo, Cd, Sb, Zn, MBC, TN, and OC had significant effects on the entire fungal community. Moreover, multiple heavy metal contaminations enhanced the complexity of the bacterial–fungal co-occurrence network, with more keystone taxa in the co-occurrence networks in the contaminated areas than in the uncontaminated areas. The keystone taxa in the contaminated area were Chloroflexi, Armatimonadetes, Proteobacteria, Acidobacteria, and Ascomycota, which were resistant to heavy metals. The keystone taxa in the uncontaminated area were Verrucomicrobia and Bacteroidetes, which were sensitive to heavy metals. In summary, soil microorganisms may resist multiple heavy metal contamination through enhanced interactions. This study could provide fundamental information for developing bioremediation mechanisms for the recovery of heavy metal-contaminated soil.

## Data availability statement

The data presented in the study are deposited in the NCBI database repository, BioProject accession numbers PRJNA979778 and PRJNA1049761.

## Author contributions

JLi: Conceptualization, Formal analysis, Investigation, Methodology, Software, Writing – original draft. QZ: Data curation, Writing – original draft. JLiu: Investigation, Visualization, Writing – original draft. SP: Investigation, Visualization, Writing – original draft. ZY: Resources, Supervision, Writing – original draft. RC: Resources, Supervision, Writing – original draft. LM: Software, Validation, Writing – original draft. JN: Conceptualization, Funding acquisition, Resources, Supervision, Writing – review & editing. TT: Conceptualization, Funding acquisition, Resources, Supervision, Writing – reviewre & editing.
