# Supplementary material for: Bacterial–fungal interactions and response to heavy metal contamination of soil in agricultural areas
Source: Front Microbiol. 2024 May 10;15:1395154. doi: 10.3389/fmicb.2024.1395154 (PMC11116572; doi:10.3389/fmicb.2024.1395154)
Supplement: Supplementary file 1 [file Table_1.DOCX]

Supplementary Material

# Supplementary Figures and Tables

## Supplementary Figures


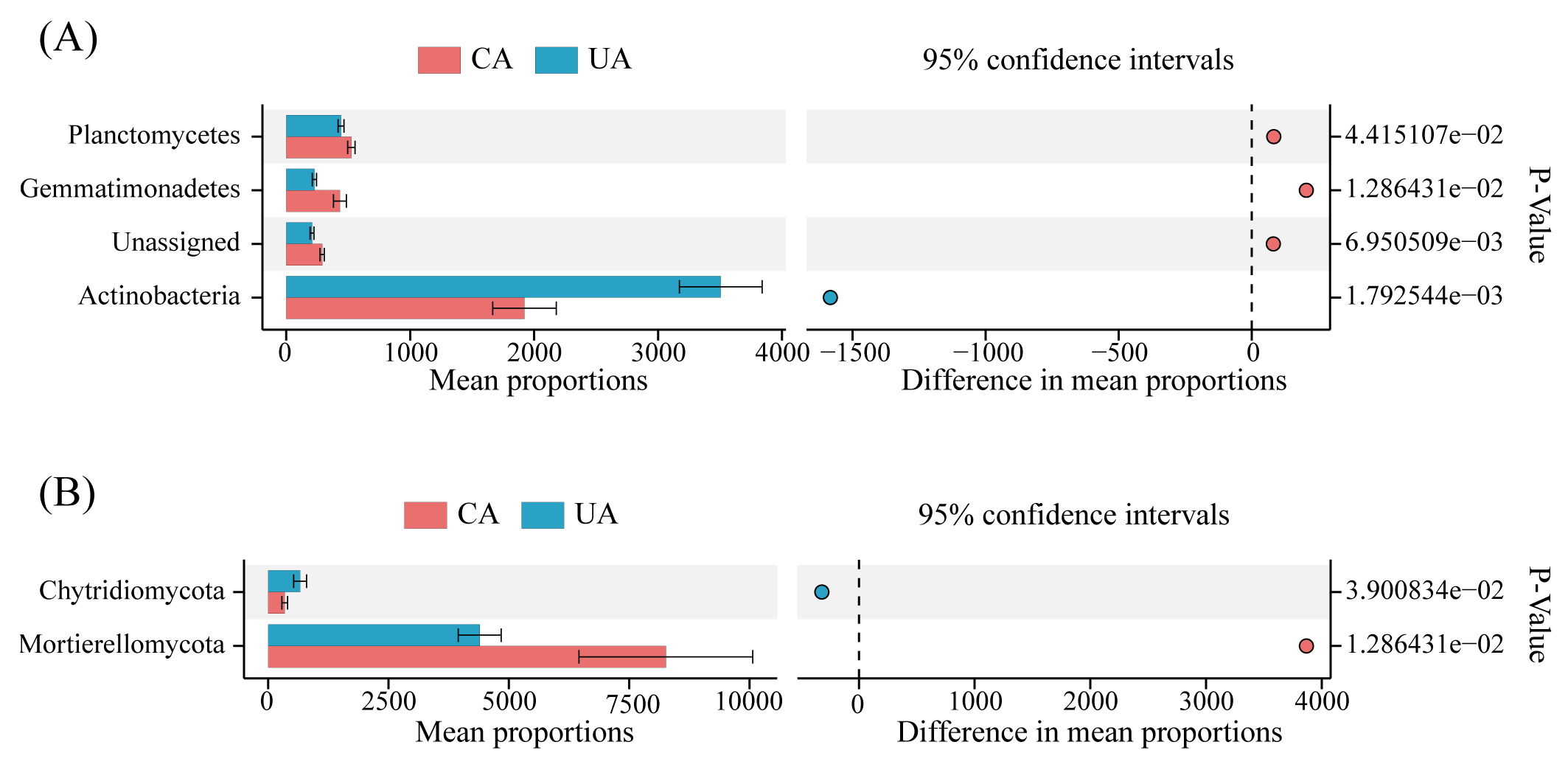


**Supplementary Figure 1.** **Soil bacterial (a) and fungal (b) relative abundance differences at phylum levels (*P*<0.05).**

## Supplementary Tables

**Table S1.** **Soil heavy metal concentrations**. Notes: P Value: the significance of heavy metal concentrations between contaminated and uncontaminated areas soils compared with two-tailed Wilcoxon rank-sum test.

| Parameter | CA | UA | P Value |
| --- | --- | --- | --- |
| Mo (ng g^-1^) | 230.14±140.72 | 86.12±28.84 | 0.001 |
| Cd (ng g^-1^) | 5463.59±10698.25 | 106.18±30.45 | 0.000 |
| Sb (ng g^-1^) | 12.50±9.96 | 3.26±1.79 | 0.001 |
| Cu (ng g^-1^) | 75056.53±59409.21 | 17997.89±2653.18 | 0.000 |
| Zn (ng g^-1^) | 377400.12±647175.64 | 47625.43±9732.57 | 0.001 |
| Hg (ng g^-1^) | 371.72±361.69 | 24.10±20.48 | 0.003 |
| Pb (ng g^-1^) | 125062.91±116930.28 | 12980.27±2181.50 | 0.000 |

**Table S2. Keystone taxa classification information (contaminated area).**

| Modular hub | Kingdom | Phylum | Genus | Zi |
| --- | --- | --- | --- | --- |
| ASV2405 | Bacteria | Chloroflexi | *-* | 3.998 |
| ASV1041 | Bacteria | Armatimonadetes | *Armatimonadetes_gp4* | 3.638 |
| ASV3755 | Bacteria | Chloroflexi | *Tepidiforma* | 3.558 |
| ASV1317 | Bacteria | Proteobacteria | *Enhydrobacter* | 2.963 |
| ASV741 | Bacteria | Acidobacteria | *Gp17* | 2.858 |
| ASV77 | Fungi | Ascomycota | *unidentified* | 2.484 |
| ASV319 | Fungi | Ascomycota | *Unassigned* | 2.484 |

**Table S3. Keystone taxa classification information (uncontaminated area).**

| Modular hub | Kingdom | Phylum | Genus | Zi |
| --- | --- | --- | --- | --- |
| ASV2082 | Bacteria | Verrucomicrobia | *Subdivision3_genera_incertae_sedis* | 2.930 |
| ASV875 | Bacteria | Bacteroidetes | *Chryseolinea* | 2.667 |
